# Mesenchymal Stem Cells and Their Extracellular Vesicles: Therapeutic Mechanisms for Blood–Spinal Cord Barrier Repair Following Spinal Cord Injury

**DOI:** 10.3390/ijms252413460

**Published:** 2024-12-16

**Authors:** Masahito Nakazaki, Takahiro Yokoyama, Karen L. Lankford, Ryosuke Hirota, Jeffery D. Kocsis, Osamu Honmou

**Affiliations:** 1 Department of Neural Regenerative Medicine, Research Institute for Frontier Medicine, Sapporo Medical University School of Medicine, Sapporo 060-8556, Hokkaido, Japan; 2 Department of Neurology, Yale University School of Medicine, New Haven, CT 06510, USA; 3 Center for Neuroscience and Regeneration Research, VA Connecticut Healthcare System, West Haven, CT 06516, USA

**Keywords:** spinal cord injury, blood–spinal cord barrier, mesenchymal stromal/stem cells, extracellular vesicles, macrophages, tight junction proteins

## Abstract

Spinal cord injury (SCI) disrupts the blood–spinal cord barrier (BSCB) exacerbating damage by allowing harmful substances and immune cells to infiltrate spinal neural tissues from the vasculature. This leads to inflammation, oxidative stress, and impaired axonal regeneration. The BSCB, essential for maintaining spinal cord homeostasis, is structurally similar to the blood–brain barrier. Its restoration is a key therapeutic target for improving outcomes in SCI. Mesenchymal stromal/stem cells (MSCs) and their secreted extracellular vesicles (MSC-EVs) have gained attention for their regenerative, immunomodulatory, and anti-inflammatory properties in promoting BSCB repair. MSCs enhance BSCB integrity by improving endothelial–pericyte association, restoring tight junction proteins, and reducing inflammation. MSC-EVs, which deliver bioactive molecules, replicate many of MSCs’ therapeutic effects, and offer a promising cell-free alternative. Preclinical studies have shown that both MSCs and MSC-EVs can reduce BSCB permeability, promote vascular stability, and support functional recovery. While MSC therapy is advancing in clinical trials, MSC-EV therapies require further optimization in terms of production, dosing, and delivery protocols. Despite these challenges, both therapeutic approaches represent significant potential for treating SCI by targeting BSCB repair and improving patient outcomes.

## 1. Introduction

### 1.1. Blood–Spinal Cord Barrier in Spinal Cord Injury

The blood–spinal cord barrier (BSCB) is a specialized component of the central nervous system (CNS) that maintains homeostasis of the spinal cord microenvironment by regulating the exchange of molecules between the blood and spinal cord tissue [1,2]. Structurally analogous to the blood–brain barrier (BBB), the BSCB in capillaries is composed of endothelial cells interconnected by tight junctions surrounded by basement membranes, pericytes, and astrocytic endfeet. These elements work synergistically to restrict the passage of potentially harmful substances while permitting the controlled entry of nutrients and essential molecules necessary for neuronal function and survival [1,2].

In the context of spinal cord injury (SCI), the integrity of the BSCB is severely compromised [3] resulting in increased permeability that allows the infiltration of inflammatory cells, blood-borne toxins, and immune mediators into the spinal cord parenchyma. In experimental models, BSCB integrity can be assessed using intravenous injection of dyes such as Evans blue which do not breach the intact BSCB [4]. BSCB disruption exacerbates the initial mechanical injury and leads to secondary injury processes including oxidative stress, excitotoxicity, and the formation of a neurotoxic environment [5]. The breakdown of the BSCB is characterized by the disassembly of tight junction proteins such as occludin, claudins, and zonula occludens-1 (ZO-1), degradation of the basal lamina, and pericyte detachment. These alterations contribute to the opening of the barrier and facilitate the development of a pro-inflammatory milieu that hinders the regenerative capacity within the spinal cord [6].

Restoration of BSCB integrity is therefore a critical target in the treatment of SCI, as it can potentially limit the extent of secondary damage and promote an environment conducive to repair and regeneration [4,7,8]. However, the complex and dynamic nature of BSCB disruption presents significant challenges in developing effective therapeutic strategies. Recent advancements in the understanding of BSCB physiology and the molecular mechanisms underlying its disruption have paved the way for novel therapeutic approaches, including the use of systemically delivered MSCs [4,8] and their secreted extracellular vesicles, MSC-EVs [7,9]. These therapeutic approaches have shown promise in stabilizing the BSCB, reducing inflammation, and supporting functional recovery in preclinical models of SCI.

### 1.2. Overview of Mesenchymal Stromal/Stem Cells and Extracellular Vesicles

MSCs are multipotent stromal cells capable of differentiating into a variety of cell types, including osteoblasts, chondrocytes, and adipocytes under appropriate conditions [10,11]. They were initially identified in bone marrow but have since been isolated from various tissues such as adipose tissue, umbilical cord blood, and placenta. MSCs are characterized by their plastic adherence in culture, expression of specific surface markers (e.g., CD73, CD90, CD105), and their ability to modulate immune responses and promote tissue repair [10,11]. In the context of regenerative medicine, MSCs have garnered significant attention due to their potent anti-inflammatory, immunomodulatory, and trophic properties [12]. These characteristics are largely mediated through paracrine signaling mechanisms rather than direct differentiation into target tissues. MSCs secrete a wide range of bioactive molecules including cytokines, growth factors, and EVs, which collectively contribute to their therapeutic effects [7,12,13].

EVs are membrane-bound particles released by cells including MSCs and generally range in size from 30 to 1000 nanometers. EVs can be classified into different subtypes, such as exosomes (30–150 nm), microvesicles (100–1000 nm), and apoptotic bodies (500–2000 nm) based on their size, biogenesis, and release pathways [14,15]. The small extracellular vesicles (sEVs) which include exosomes have gained much interest in the field of regenerative medicine due to their ability to transfer bioactive cargo including proteins, lipids, mRNAs, and microRNAs to recipient cells [16,17]. Exosomes, in particular, are characterized as a subset of sEVs in the 30–150 nm range and are enriched in sphingolipids and tetraspanin proteins such as CD63, CD9, and CD81 [18]. These vesicles are generated through the endosomal pathway, where multivesicular bodies fuse with the plasma membrane releasing exosomes into the extracellular space [19]. Exosomes carry a variety of molecular cargo that can modulate the function of target cells contributing to their therapeutic potential [19]. MSC-derived EVs (MSC-EVs) are considered a key component of the MSC secretome and have been shown to recapitulate many of the therapeutic effects of MSCs themselves through exosomes within the MSC-EV population [7]. This includes modulation of the immune response, promotion of angiogenesis [20], stabilization of the BSCB [4], inhibition of apoptosis [21] and enhancement of tissue repair [7,22].

The therapeutic potential of MSC-EVs is particularly relevant in the context of neurological disorders including SCI, where their ability to cross biological barriers and deliver therapeutic molecules to target cells offers significant promise. Recent studies have demonstrated that MSC-EVs can repair BSCB dysfunction, reduce neuroinflammation, and promote neuroprotection and regeneration. These findings suggest that MSC-EVs could serve as a powerful alternative or adjunct to traditional MSC-based therapies [7,18,23,24].

### 1.3. Objectives of the Review

The primary objective of this review is to explore the therapeutic potential of MSCs and their derived EVs in the context of BSCB repair following SCI. While both MSC and MSC-EV therapies have demonstrated significant promise in promoting neuroprotection, modulating immune responses, and enhancing tissue regeneration, their interrelated roles and complementary mechanisms of action in BSCB repair require further elucidation. This review aims to provide a comprehensive understanding of how MSCs and MSC-EVs contribute to the restoration of BSCB integrity and function. By examining the current evidence, we will discuss the biological processes through which MSCs exert their effects, including the secretion of bioactive molecules and EVs. We will also explore how MSC-EVs, as a critical component of the MSC secretome, replicate and possibly enhance the therapeutic outcomes of MSC-based therapies. This review will provide insights into the interconnected roles of MSCs and MSC-EVs in the repair of the blood–spinal cord barrier, emphasizing their relevance in developing effective treatments for spinal cord injury and related neurological conditions.

## 2. The Blood–Spinal Cord Barrier in Health and Disease

### 2.1. Structure and Function of the BSCB

**Figure 1:**
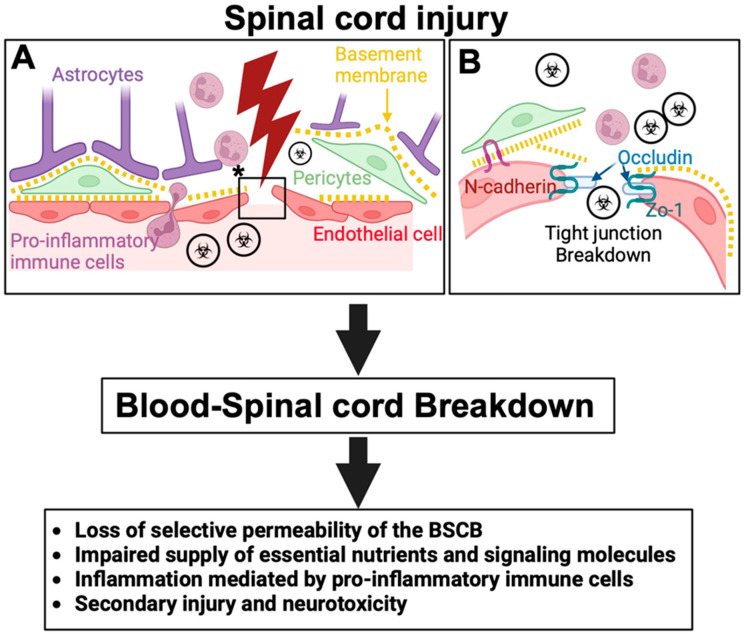
Blood–Spinal Cord Barrier Disruption Following Spinal Cord Injury (SCI).(**A**) Schematic illustration depicting the cellular components of the blood–spinal cord barrier (BSCB) under normal conditions, with astrocytes, pericytes, and endothelial cells maintaining BSCB integrity. Following SCI, the barrier is disrupted, allowing infiltration of harmful substances such as pathogens, pro-inflammatory cells, and neurotoxic molecules into the spinal cord parenchyma. The area marked with an asterisk (*) in panel (**A**) is shown in an enlarged view in panel (**B**). (**B**) Tight junction breakdown is shown, where key proteins such as N-cadherin, occludin, and ZO-1 dissociate, resulting in increased BSCB permeability. This disruption leads to a loss of selective permeability, impaired nutrient and signaling molecule exchange, enlargement of blood vessels, neuroinflammation, and secondary injury processes that exacerbate neurotoxicity.

The BSCB is a highly specialized vascular interface that plays a crucial role in maintaining the homeostasis of the spinal cord microenvironment. Analogous to the BBB in the brain, the BSCB regulates the passage of substances between the blood and the spinal cord, ensuring a stable milieu that is essential for proper neuronal function and protection from potentially harmful agents [1,2]. Structurally, the BSCB is composed of several key components that work in concert to restrict paracellular and transcellular transport. Tight junction proteins including the claudins, occludins, ZO-1, and N-cadherin are essential for maintaining the structural integrity of the BSCB. Occludin expression may be a crucial determinant of the tight junction permeability properties of endothelial cells in different tissues [25]. ZO-1 serves as a scaffolding protein linking transmembrane tight junction proteins to the cytoskeleton, thereby stabilizing the barrier [26,27]. The calcium-dependent adhesion molecule N-cadherin plays a crucial role in maintaining the adhesion between pericytes and endothelial cells, which is essential for vascular stability and integrity [28]. N-cadherin forms adherens junctions which act in concert with tight junctions to facilitate strong cell–cell adhesions. In the BSCB, N-cadherin mediates the attachment of pericytes to endothelial cells, contributing to the structural integrity of blood vessels. This adhesion is critical for maintaining the barrier function and regulating vascular permeability. Disruption in the expression and localization of these proteins following SCI leads to increased permeability of the BSCB, worsening the extent of tissue damage (Figure 1).

Pericytes regulate capillary diameter and control blood flow while also maintaining endothelial cell function [29,30,31,32]. In normal intact CNS tissue pericytes wrap around endothelial cells providing structural and functional support to the BSCB. This coverage is essential for regulating endothelial cell function including tight junction maintenance and vessel permeability [4,8]. Pericytes also contribute to the stability of the tight junctions [33] (Figure 1). Together, endothelial cells and pericytes maintain the barrier between the brain parenchyma and the rest of the body and they play critical roles in repair following CNS injury [8,29,34,35]. Astrocytic endfeet, which are extensions of astrocytes that envelop the blood vessels, surround the basal lamina of the structure, and are essential for maintaining the ionic balance and supplying metabolic support to neurons [36]. Astrocytes also secrete signaling molecules that influence the function of endothelial cells and pericytes, thereby modulating BSCB permeability [37].

The selective permeability of the BSCB is vital for protecting spinal cord neurons from fluctuations in blood composition and exposure to neurotoxic substances. It allows the controlled entry of essential nutrients, ions, and signaling molecules while preventing the infiltration of large molecules, pathogens, and immune cells that could trigger inflammatory responses or damage neural tissue. The BSCB allows the exchange of certain molecules such as glucose, amino acids, and lipophilic substances through specific transport mechanisms [38] and contains active transport systems, including efflux pumps like P-glycoprotein, which help to remove potentially harmful substances from the spinal cord parenchyma [39].

Understanding the complex structure and function of the BSCB is critical for developing therapeutic strategies aimed at repairing or restoring barrier integrity following SCI. The BSCB’s ability to regulate the spinal cord microenvironment is indispensable for both normal spinal cord function and the recovery process following injury. The trajectory of disruption and recovery of the BSCB correlates with functional loss and recovery after SCI [4]. Disruption of this barrier after SCI can lead to detrimental outcomes including increased permeability, inflammation, and secondary neuronal damage highlighting the importance of therapeutic approaches that can restore BSCB function [3,4,8].

### 2.2. Mechanisms of BSCB Disruption in Spinal Cord Injury

SCI induces both primary and secondary injury mechanisms that lead to the disruption of the BSCB, a critical protective structure in maintaining spinal cord homeostasis [3]. SCI leads to the disruption of endothelial cells resulting in the breakdown of tight junctions and increased barrier permeability [7]. This breakdown allows for the infiltration of blood-borne molecules, immune cells, and toxins into the spinal cord parenchyma exacerbating inflammation and oxidative stress (Figure 1). Moreover, endothelial cell damage is further associated with the upregulation of matrix metalloproteinases (MMPs), such as MMP-9 [40], which degrade the extracellular matrix and junctional proteins contributing to BSCB breakdown.

Pericytes are also impacted by SCI. After SCI, pericytes dissociate from endothelial cells and migrate into areas of glial scarring near the lesion epicenter resulting in a loss of pericyte–endothelial cell interaction [4,29,41]. This dissociation not only disrupts vessel stability but also contributes to the enlargement (engorgement) of blood vessels and further leakage through the compromised BSCB [4]. Over time, the number of pericytes increases within scar tissue, but this does not necessarily correlate with improved barrier function [42]. While the quantity of pericytes in the injury site is important, their ability to appropriately interact with and wrap around endothelial cells and maintain a high coverage rate is critical for preserving BSCB integrity [4,8].

After SCI, the disruption of the BSCB allows activated macrophages to infiltrate the injury site, releasing pro-inflammatory cytokines such as tumor necrosis factor-alpha (TNF-α) and interleukin-1 beta (IL-1β) [43]. These cytokines further contribute to BSCB breakdown by weakening endothelial tight junctions and promoting the degradation of the extracellular matrix [44]. Macrophages, particularly the pro-inflammatory M1 phenotype, also produce reactive oxygen species (ROS) and nitric oxide (NO), which further damage the endothelial barrier and promote the progression of secondary injury [45]. However, macrophages can also adopt an anti-inflammatory M2 phenotype, which is associated with tissue repair and the resolution of inflammation. M2 macrophages secrete factors that support endothelial cell survival, promote tight junction repair and potentially enhance pericyte–endothelial interactions [46,47,48]. Therefore, therapies that shift macrophage polarization towards reducing the harmful activity of M1 macrophages and promoting the reparative functions of M2 macrophages could be pivotal in restoring and stabilizing the BSCB after injury.

## 3. MSC-Based Therapy for SCI Targeting the BSCB: Cellular vs. Extracellular Vesicle Therapy

MSC therapy has emerged as a promising approach for treating SCI, particularly by targeting the BSCB. MSCs offer a multifaceted therapeutic approach to repair and stabilize the BSCB, thereby mitigating further damage and promoting functional recovery [4]. While the preliminary results of MSC therapies have been promising, growing attention is also being directed towards EV therapy, a cell-free approach, as a complementary method to deliver MSC-based treatments to a broader patient population. MSC-EVs have garnered significant attention due to their ability to mediate many of the same regenerative and immunomodulatory effects as MSCs [7]. EVs are lipid bilayer-enclosed particles secreted by cells, and they play essential roles in intercellular communication by transferring proteins, lipids, RNAs, and other bioactive molecules to target cells. The biogenesis and characteristics of MSC-EVs are of critical importance to understanding their therapeutic potential, particularly in the context of SCI and BSCB repair. In this section, we review research conducted using MSC and MSC-EV as therapeutic agents in SCI, focusing particularly on their role in repairing the BSCB.

### 3.1. Restoration of BSCB Function by MSC Therapy

Several studies have reported the use of MSCs to improve BSCB disruption following SCI [4,7,8]. Matsushita et al. (2015) explored the therapeutic effects of intravenous infusion of MSCs in a rat model of SCI [4]. Specifically, they examined how intravenous infusion of MSCs influences the vascular architecture and BSCB, and locomotor recovery over a 10-week period following a contusive SCI. The findings reveal that SCI induces widespread disruption of the BSCB resulting in increased vascular permeability and changes in the microvascular structure. This disruption of the BSCB was persistent and extended rostrally and caudally beyond the lesion site. Following MSC infusion, there was a significant reduction in BSCB permeability, which correlated with improved locomotor function starting one week after cell administration. Although the infused MSCs did not migrate to the spinal cord, their presence in the lungs appeared to trigger a systemic effect that contributed to the repair of the BSCB. The study suggests that the therapeutic effect of MSCs may be due to the secretion of certain factors that modulate gene expression in endothelial cells, thereby enhancing the recovery of vascular integrity. Therapeutic effects on BSCB were also observed in a chronic SCI model. Morita et al. (2016), analyzed the changes in the BSCB in a model of SCI following intravenous infusion of MSCs [8]. MSCs were administered 10 weeks after inducing severe contusive SCI. The researchers found that MSC treatment significantly reduced BSCB leakage, as assessed by the extravasation of Evans blue dye. This suggests that MSCs helped restore the integrity of the BSCB in chronically injured spinal cords. These findings indicate that MSCs contributed to the stabilization of the BSCB and reduced vascular permeability, which may have played a role in the observed functional improvements following SCI.

The therapeutic effects of MSCs on the BSCB and BBB have been widely reported in various pathological conditions beyond SCI. For example, in stroke models, MSCs have been shown to reduce BBB permeability, minimize infarct size, and promote neurovascular recovery [40,49,50]. In a model of amyotrophic lateral sclerosis (ALS), studies have demonstrated that MSCs can help preserve BBB integrity, potentially delaying the progression of neurodegeneration by preventing inflammatory cells from entering the central nervous system [51]. Similarly, in models of dementia MSCs have been reported to restore BBB function by reducing amyloid-beta accumulation and associated neuroinflammation [31,52]. These findings collectively reinforce the idea that MSCs exert broad protective effects on both the BSCB and BBB across a range of neurological disorders, supporting their potential as a therapeutic option for maintaining or restoring barrier function in various disease contexts.

### 3.2. Recovery of BSCB Function via MSC-EV Therapy

MSC-EVs are pivotal mediators in MSC therapy and their considerable advantages for clinical application have driven extensive research aimed at restoring BSCB function after SCI [7,53,54]. Lu et al. (2019) explored the therapeutic potential of MSC-EVs in restoring the integrity of the BSCB after SCI [53]. MSC-EVs were administered via tail vein injections at 30 min and 24 h post-injury and the results showed that MSC-EV administration significantly reduced the permeability of the BSCB as indicated by decreased leakage of Evans blue dye into the spinal cord tissue. The early administration of MSC-EVs following SCI appears to play a crucial role in protecting the BSCB, promoting vascular integrity, and contributing to functional recovery in this rat model of SCI. Wang et al. (2021) also investigated the role of MSC-EVs in restoring the integrity of the BSCB following acute spinal cord injury SCI in a rat model [54]. MSC-EVs were administered immediately after SCI, with subsequent daily subcutaneous treatments for a week near the site of injury. The results demonstrated that MSC-EVs significantly reduced BSCB permeability as evidenced by reduced leakage of Evans blue dye and FITC-dextran. Nakazaki et al. (2021) reported the therapeutic effects of MSC-EVs on the BSCB following severe SCI in a rodent model [7]. In this study, MSC-sEVs were administered intravenously at 7 days post-SCI over 3 consecutive days. They found that MSC-sEVs significantly reduced BSCB leakage, as demonstrated by reduced Evans blue dye extravasation at 14 days post-SCI. These studies collectively highlight the crucial role of MSC-EVs in stabilizing the BSCB after SCI. Whether administered immediately or delayed after injury, MSC-EVs consistently demonstrate their ability to reduce BSCB permeability, enhance vascular integrity, and ultimately support functional recovery. These findings underscore the therapeutic potential of MSC-EVs, offering a noncellular and systemically delivered approach to target BSCB repair and neuroprotection.

## 4. Mechanisms of MSC-Based Therapy for SCI Targeting the BSCB

### 4.1. Increase in Endothelial Cell Coverage by Pericytes

**Figure 2:**
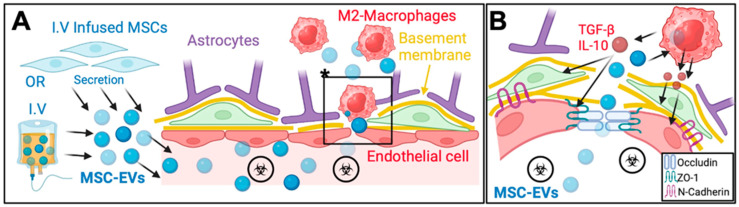
MSC and MSC-EV Mediated Repair of the Blood–Spinal Cord Barrier Post-SCI. (**A**). Intravenous (I.V) infusion of mesenchymal stem cells (MSCs) or their extracellular vesicles (MSC-EVs) plays a pivotal role in repairing the blood–spinal cord barrier (BSCB) following spinal cord injury (SCI). MSC-EVs specifically target M2 macrophages, enhancing their anti-inflammatory and reparative functions. This leads to the secretion of bioactive molecules that aid in endothelial cell stabilization and reduce inflammation at the injury site. The area marked with an asterisk (*) in panel (**A**) is shown in an enlarged view in panel (**B**). (**B**). MSC-EVs promote the restoration of the BSCB by upregulating tight junction proteins, such as occludin, ZO-1, and N-cadherin. These proteins help re-establish tight junction integrity, improving vascular stabilization and reducing the infiltration of harmful molecules into the spinal cord. Anti-inflammatory cytokines like TGF-β and IL-10, secreted by M2 macrophages, further support long-term BSCB repair and functional recovery.

The endothelial cell–pericyte interaction is vital for the structural and functional maintenance of the BSCB. MSC transplantation has been shown to increase the coverage of endothelial cells by pericytes [4,8,31,55] (Figure 2). After SCI, the dissociation of pericytes from endothelial cells contributes to the breakdown of the BSCB [1]. MSCs exert their effects through the secretion of bioactive molecules that stimulate pericyte recruitment and proliferation. Growth factors such as Transforming Growth Factor-beta (TGF-β) and angiopoietin secreted by MSCs, promote the migration of pericytes to the injury site and increase co-association of pericytes with endothelial cells [31,50]. Furthermore, MSCs have been shown to upregulate proteins such as N-cadherin, which is crucial for the adhesion between pericytes and endothelial cells [28]. This improved endothelial cell–pericyte coverage helps to maintain vascular stability and prevent ongoing barrier leakage after SCI. Menezes et al. (2020) reported that MSCs enhanced spinal cord injury recovery by recruiting pericytes [55]. Importantly, the study showed that the presence of MSCs was necessary for the full maturation of blood vessels. While conditioned medium promoted angiogenesis and increased vascular density, it failed to recruit pericytes effectively or support the maturation of blood vessels, resulting in vascular instability and hemorrhagic areas. In contrast, animals treated with MSCs exhibited a significant increase in pericyte coverage on the vascular walls, which was associated with improved blood vessel maturation and integrity of the BSCB.

MSC-EVs have also been shown to enhance endothelial cell–pericyte coverage similar to MSC treatment. By promoting this critical association, MSC-EVs contribute to the stabilization of the BSCB and reduce vascular permeability following spinal cord injury, further supporting their therapeutic potential in vascular repair [53,56]. Lu et al. (2019) reported that MSC-EVs help prevent the detachment of pericytes from the vascular wall, thus maintaining the structural integrity of the BSCB. By promoting pericyte recruitment and reducing pericyte migration, BMSC-EVs preserved vascular stability [53].

It was suggested that MSC-EVs inhibited pericyte migration from endothelial cells via suppression of the nuclear factor-kappa B (NF-κB) signaling pathway. This inhibition of pericyte migration contributed to a decrease in BSCB permeability, which ultimately reduced inflammation and supported neuronal survival. Zhou et al. (2022) suggested that the improvement in the endothelial cell–pericyte ratio by MSC-EVs is due to the inhibition of pericyte pyroptosis, a form of programmed inflammatory cell death [56]. This study highlights the role of MSC-EVs in protecting the BSCB and preventing pericyte pyroptosis in a rat model of SCI. MSC-EVs were administered shortly after injury, significantly increasing pericyte–endothelial cell coverage on the vascular walls, thereby improving BSCB integrity. In vitro, MSC-EVs decreased caspase-1 expression and reduced the release of interleukin-1β (IL-1β), both key mediators of pyroptosis. This protective action not only improved pericyte survival but also maintained the BSCB’s structural integrity, helping to reduce inflammation and support neuronal survival.

Both MSCs and MSC-EVs have been shown to enhance endothelial cell–pericyte coverage, which is crucial for maintaining the integrity of the BSCB following SCI. By recruiting pericytes and promoting their interaction with endothelial cells, MSCs, and MSC-EVs stabilize blood vessels, reduce vascular permeability, and reduce ongoing microvasculature leakage. This improved vascular stability supports tissue repair and functional recovery. Additionally, MSC-EVs prevent pericyte pyroptosis, further enhancing pericyte survival and BSCB integrity, underscoring their therapeutic potential in vascular repair and neuroprotection after SCI.

### 4.2. Restoration of Tight Junction Integrity

In addition to enhancing endothelial cell–pericyte coverage, MSCs and MSC-EVs play a pivotal role in restoring the integrity of tight junctions within the BSCB following SCI. Tight junction proteins, such as occludin, claudin-5, and ZO-1, are essential components of the BSCB that regulate vascular permeability and maintain the barrier’s selective permeability. Following SCI, the expression of these proteins is often downregulated, contributing to increased leakage across the BSCB and exacerbating tissue damage.

MSC transplantation upregulates the expression of tight junction proteins, thereby restoring the structural integrity of the BSCB [7,52,57]. This restoration is believed to occur through the paracrine signaling effects of MSCs, where the secretion of factors such as vascular endothelial growth factor (VEGF) and angiopoietin-1 contributes to the repair and strengthening of tight junctions. Park et al. (2015) reported that MSC therapy effectively restores tight junction integrity and BBB stability [52]. By modulating VEGF-A levels, MSCs inhibit the activation of endothelial nitric oxide synthase (eNOS) preventing the downregulation of tight junction proteins. This allows the tight junction proteins such as claudin-5 to recover, thus reinforcing the integrity of the BBB. Additionally, MSCs exert anti-inflammatory effects by reducing pro-inflammatory cytokines such as IL-1β, which further diminishes VEGF-A levels in astrocytes. This cascade of cellular events reduces BBB permeability and helps restore its protective function, providing potential therapeutic benefits in neurodegenerative diseases such as Parkinson’s disease.

Chen et al. (2015) demonstrated that MSCs upregulate ZO-1 and claudin-5 through the secretion of TNF-α stimulated gene/protein 6 (TSG-6) [57]. TSG-6 has potent anti-inflammatory properties that allow MSCs to suppress the NF-κB signaling pathway, which is typically activated in response to inflammation and oxidative stress [57,58,59,60]. By inhibiting NF-κB, MSCs reduce the production of pro-inflammatory cytokines like TNF-α and IL-1β, which are known to degrade tight junction proteins and disrupt BBB integrity. The suppression of NF-κB thus prevents the inflammatory cascade that would otherwise lead to the downregulation of ZO-1 and claudin-5. Nakazaki et al. (2021) provided further insight into how MSC therapy reinforces tight junction integrity. MSC infusion significantly upregulated the expression of tight junction proteins such as occludin, N-cadherin, and ZO-1, which are crucial for BSCB function [7]. Levels of mRNA and protein for these tight junction proteins were increased in MSC-treated SCI rats compared to untreated SCI rats, particularly at 14 days post-SCI. These changes resulted in reduced microvascular leakage and increases in ZO-1, occludin, and N-cadherin protein levels were sustained for at least 70 days post-SCI, suggesting that MSC therapy can produce long-term stabilization of the BSCB. This restoration of tight junctions plays a vital role in reducing neuroinflammation and tissue damage, ultimately promoting functional recovery after SCI.

Similarly, MSC-EVs have been shown to enhance tight junction integrity. MSC-EVs play a crucial role in upregulating tight junction proteins like ZO-1 and occludin, which are essential for maintaining the integrity of barriers such as the blood–brain and blood–spinal cord barrier. MSC-sEVs achieve this through a series of targeted cellular interactions and signaling cascades. Nakazaki et al. (2021) suggested that upon release or intravenous injection, MSC-sEVs were taken up by M2 macrophages at sites of spinal cord injury [7]. These M2 macrophages known for their tissue-repairing and anti-inflammatory functions [46] increased in number at the lesion site, and the expression levels of tight junction proteins, including ZO-1, occludin, and N-cadherin were upregulated by the MSC-EV treatment [7] (Figure 2). Lu et al. (2019) reported that the mechanism behind the protective effect involves the TIMP2/MMP pathway. Intravenous infusion of MSC-EVs increased the expression of Tissue Inhibitor of Metalloproteinases-2 (TIMP2), which inhibits matrix metalloproteinases (MMP-2 and MMP-9), enzymes responsible for breaking down extracellular matrix components and tight junction proteins [53]. By inhibiting these MMPs, MSC-EVs preserved essential junction proteins such as claudin-5, occludin, and ZO-1, which are crucial for maintaining BSCB integrity. When TIMP2 expression was knocked down in the MSC-EVs, the protective effect on the BSCB was significantly diminished, resulting in increased barrier permeability and reduced recovery. Using a rodent model of stroke, Qiu et al. (2022) presented compelling evidence demonstrating that MSC-EVs enhance the expression of tight junction proteins, particularly ZO-1, and occludin, by specifically inhibiting the TLR4/NF-κB signaling pathway [61]. After ischemic stroke, tissue plasminogen activator (tPA) treatment can lead to BBB disruption, primarily by triggering inflammation and the activation of astrocytes. This inflammatory response disrupts tight junctions, weakening the integrity of the BBB. MSC-EVs enriched with miR-125b-5p are taken up by astrocytes in the injured brain. This microRNA plays a crucial role by inhibiting the activation of Toll-like receptor 4 (TLR4) and subsequently blocking the NF-κB pathway in astrocytes. The inhibition of NF-κB prevents the transcription of pro-inflammatory cytokines, which would otherwise contribute to the degradation of tight junction proteins like ZO-1 and occludin. By downregulating the TLR4/NF-κB signaling, MSC-EVs not only reduce inflammation but also promote the restoration and upregulation of ZO-1 and occludin. This process strengthens the tight junctions between endothelial cells, ultimately restoring BBB integrity and reducing permeability [61].

Overall, both MSC and MSC-EV therapies have demonstrated significant potential in promoting BSCB repair by upregulating tight junction proteins. The sustained expression of these proteins after treatment highlights the long-term benefits of these therapies in maintaining BSCB integrity and facilitating functional recovery.

### 4.3. Immunomodulation and Reduction in Inflammation

A key mechanism by which MSCs exert therapeutic effects in SCI is through their potent immunomodulatory properties, which directly impact the BSCB [62,63,64]. Following SCI, the disruption of the BSCB leads to the infiltration of inflammatory cells including neutrophils, macrophages, and lymphocytes exacerbating secondary damage through the release of pro-inflammatory cytokines such as TNF-α, IL-1β, and IL-6 [63,65]. This inflammatory response contributes to the further degradation of tight junction proteins and increased BSCB permeability worsening tissue damage. Therefore, immunomodulation becomes crucial. MSCs modulate the immune response by releasing anti-inflammatory factors and regulating immune cell behavior [65,66]. These factors secreted by MSCs can vary based on the pathological conditions they encounter, influencing key immune cells, including T cells, dendritic cells, and macrophages. MSCs release various biologically active molecules such as TGF-β, prostaglandin E2 (PGE2), hepatocyte growth factor (HGF) and EVs, which mediate their immunomodulatory functions. For example, TGF-β plays a significant role in suppressing T-cell proliferation and promoting the expansion of regulatory T cells [67]. PGE2, another key factor, is involved in shifting the immune response from a pro-inflammatory state to a more anti-inflammatory state, affecting macrophage polarization and dendritic cell maturation [68]. MSC transplantation modified the post-injury inflammatory environment, particularly by promoting a shift in macrophage activation [65,69]. MSC transplantation (direct intraspinal injection into the lesion) was shown to promote the alternative activation of macrophages into an anti-inflammatory (M2) phenotype. This phenotypic shift was accompanied by an increase in anti-inflammatory cytokines, such as IL-4 and IL-13, and a decrease in pro-inflammatory markers like TNF-α and IL-6 [65]. These mechanisms are crucial in the therapeutic applications of MSCs in immune-related diseases [66].

MSC-EVs have also been shown to contribute to immunomodulation. EVs contain bioactive molecules such as microRNAs, proteins, and lipids that target specific immune pathways, dampening the inflammatory response [53,70,71]. For instance, MSC-EVs have been reported to downregulate pro-inflammatory cytokine expression and inhibit the activation of NF-κB, a key regulator of inflammation [53]. This reduction in inflammatory signaling helps to protect the BSCB, preventing further damage and promoting its repair. Recent studies have shown that MSC-EVs have strong immunomodulatory effects, primarily by delivering therapeutic RNAs and proteins [70,71,72]. Kim et al. (2020) using proteomics and microRNA sequencing, identified key molecules (TGF-β1, pentraxin 3 (PTX3), let-7b-5p, and miR-21-5p) that were enriched in the MSC-EVs and played significant roles in immunomodulation [71]. Additional research has revealed that MSC-EVs have emerged as potent immunomodulators, particularly through their interactions with macrophages. One of the most important mechanisms through which MSC-EVs modulate macrophage function is by delivering specific miRNAs that target key components of inflammatory signaling pathways [70,72,73]. For example, miR-182-5p, found within MSC-EVs, plays a crucial role in downregulating the Toll-like receptor 4 (TLR4) signaling pathway in macrophages [72]. TLR4 is responsible for recognizing pathogen-associated molecular patterns, such as bacterial lipopolysaccharides (LPS), which trigger the activation of NF-κB, a major transcription factor that promotes the expression of pro-inflammatory cytokines. By suppressing TLR4 and its downstream signaling molecules like MyD88 and NF-κB, miR-182-5p reduces the inflammatory response in macrophages, leading to a shift toward the M2 anti-inflammatory state. Wang et al. (2015) demonstrated that MSC-EVs containing miR-223-5p and -3p from wild-type mice reduced LPS-induced cytokine production in macrophages, dampened systemic inflammation, and improved survival in a murine sepsis model [70]. Another significant miRNA found in MSC-EVs is miR-146a, which is often enriched in MSC-EVs under inflammatory conditions, and also plays a key role in modulating macrophage activity [73]. This miRNA targets important signaling molecules such as IRAK1 (Interleukin-1 Receptor-Associated Kinase 1) and TRAF6 (TNF Receptor-Associated Factor 6), both of which are involved in the TLR-mediated activation of NF-κB. By inhibiting these molecules, miR-146a effectively reduces the pro-inflammatory signaling cascades within macrophages. Studies have shown that MSC-EVs containing miR-146a are particularly effective in promoting the M2 phenotype, characterized by increased expression of anti-inflammatory cytokines such as IL-10 and reduced expression of pro-inflammatory markers. These miRNAs work together to reprogram macrophages toward an anti-inflammatory state, contributing to the resolution of inflammation, thus preventing inflammatory response contributing to further degradation of tight junction proteins and increased BSCB permeability. In this regard, labeled MSC-EVs intravenously infused into a spinal cord injured rat specifically target M2 type macrophages in the lesion [18].

## 5. MSC and MSC-EV Therapies: A Comparison in SCI Treatment

MSCs and their secreted EVs both show promising therapeutic potential in treating SCI, particularly in repairing the BSCB through restoration of tight junction proteins and modulating immune responses. However, despite these commonalities, MSC transplantation and MSC-EV therapy differ significantly in their modes of delivery and therapeutic dynamics. MSC transplantation involves the direct infusion of live cells into the body, where these cells can potentially engraft in the injured tissue or other organs [4] continuously secreting EVs and other paracrine factors over an extended period. This ongoing release of bioactive molecules allows MSCs to exert prolonged effects on the injury site. In contrast, MSC-EVs are pre-collected vesicles administered as a concentrated therapeutic product. While MSC-EVs can replicate many of the effects seen with MSCs, they have a limited lifespan in circulation [18], necessitating multiple dosing over several days to mimic the sustained paracrine signaling observed with live MSCs [7].

Moreover, there are differences in the clinical data available for the two therapies. MSC therapy has been the subject of numerous clinical trials, many of which have demonstrated its safety and potential efficacy, with some reports suggesting significant improvements in patients [74], although larger studies are still needed to fully establish its effectiveness. In contrast, MSC-EV therapy is still in its early stages of clinical research. A critical limitation in the current landscape of MSC-EV therapy is the lack of robust protocols for scalable and reproducible production [75]. Large-scale manufacturing processes must not only ensure sufficient yield but also maintain batch-to-batch consistency in quality and potency, which are essential for achieving reliable therapeutic outcomes. Another pressing issue is the need for standardized dosing regimens. Currently, the optimal dosage for MSC-EVs in different patient populations and disease severities remains unclear. This uncertainty complicates the ability to predict consistent therapeutic efficacy across diverse clinical settings. Furthermore, advanced delivery methods are required to enhance EV targeting and retention at the injury site. While preclinical studies suggest low immunogenicity, the long-term safety profile of MSC-EVs has not been comprehensively evaluated. Potential risks, such as off-target effects and accumulation in non-target tissues, highlight the need for more thorough investigations in large-scale clinical trials. These trials must include rigorous monitoring protocols to ensure both immediate and long-term safety. Larger scale clinical trials to determine efficacy and more thorough safety checks of MSC-EV therapy are necessary before it can be widely applied clinically. While both MSC-cellular and EV therapeutic approaches show great promise for SCI treatment, MSC-EVs face additional challenges in terms of clinical validation and optimization for therapeutic use. The disparities in clinical evidence highlight the need for extensive investigations to comprehensively evaluate the efficacy of both modalities. Comparative studies focusing on therapeutic outcomes and underlying mechanisms are essential to identify optimal applications for each approach and guide future clinical strategies.

## 6. Conclusions

In conclusion, both mesenchymal stromal/stem cells (MSCs) and their extracellular vesicles (MSC-EVs) hold significant promise for treating SCI, particularly by reducing inflammation and restoring blood–spinal cord barrier (BSCB) integrity. MSCs enhance vascular stability, reinforce tight junctions, and modulate immune responses contributing to BSCB repair and improved recovery. Systemic MSC-EV treatment, as a cell-free therapy, offers similar benefits by delivering therapeutic molecules directly to damaged tissues without potential issues related to live cell delivery. While MSC therapies are further along in clinical trials, MSC-EV therapies require more research to optimize their large-scale production, dosing, and delivery protocols. Despite these challenges, both approaches show great potential for advancing SCI treatment by promoting BSCB repair and functional recovery.
